# SCO-spondin from embryonic cerebrospinal fluid is required for neurogenesis during early brain development

**DOI:** 10.3389/fncel.2013.00080

**Published:** 2013-06-03

**Authors:** A. Vera, K. Stanic, H. Montecinos, M. Torrejón, S. Marcellini, T. Caprile

**Affiliations:** Department of Cell Biology, Faculty of Biological Sciences, University of ConcepciónBiobío Region, Chile

**Keywords:** SCO-spondin, cerebrospinal fluid, neuroepithelium, neurogenesis, posterior commissure, mesencephalon, subcommissural organ

## Abstract

The central nervous system (CNS) develops from the neural tube, a hollow structure filled with embryonic cerebrospinal fluid (eCSF) and surrounded by neuroepithelial cells. Several lines of evidence suggest that the eCSF contains diffusible factors regulating the survival, proliferation, and differentiation of the neuroepithelium, although these factors are only beginning to be uncovered. One possible candidate as eCSF morphogenetic molecule is SCO-spondin, a large glycoprotein whose secretion by the diencephalic roof plate starts at early developmental stages. *In vitro*, SCO-spondin promotes neuronal survival and differentiation, but its *in vivo* function still remains to be elucidated. Here we performed *in vivo* loss of function experiments for SCO-spondin during early brain development by injecting and electroporating a specific shRNA expression vector into the neural tube of chick embryos. We show that SCO-spondin knock down induces an increase in neuroepithelial cells proliferation concomitantly with a decrease in cellular differentiation toward neuronal lineages, leading to hyperplasia in both the diencephalon and the mesencephalon. In addition, SCO-spondin is required for the correct morphogenesis of the posterior commissure and pineal gland. Because SCO-spondin is secreted by the diencephalon, we sought to corroborate the long-range function of this protein *in vitro* by performing gain and loss of function experiments on mesencephalic explants. We find that culture medium enriched in SCO-spondin causes an increased neurodifferentiation of explanted mesencephalic region. Conversely, inhibitory antibodies against SCO-spondin cause a reduction in neurodifferentiation and an increase of mitosis when such explants are cultured in eCSF. Our results suggest that SCO-spondin is a crucial eCSF diffusible factor regulating the balance between proliferation and differentiation of the brain neuroepithelial cells.

## Introduction

After the closure of the anterior neuropore, the cranial region of the neural tube enlarges and generates the encephalic vesicles. During early development stages, these vesicles are delineated by the neuroepithelium, a pseudostratified epithelium that will eventually generate all the neurons and glial cells of the anterior adult central nervous system (CNS). The brain vesicles are filled with embryonic cerebrospinal fluid (eCSF), which plays important roles in encephalic development at both embryonic and fetal stages, regulating the survival, proliferation, and neural differentiation of the neuroepithelial progenitor cells (Gato et al., 2005; Salehi and Mashayekhi, 2006; Gato and Desmond, 2009; Zappaterra and Lehtinen, 2012). It has been proposed that the eCSF exerts its function by controlling neuroepithelial proliferation in response to internal liquid pressure (Desmond et al., 2005) and by facilitating the interaction of diffusible factors with the neuroepithelial cells apical surface (Gato and Desmond, 2009). The existence and importance of such diffusible factors have been demonstrated *in vitro* on mesencephalic and cortical explants, which develop normally in the presence of eCSF, whereas the absence of eCSF causes a decrease in neuroepithelial cells proliferation, differentiation, and survival (Gato et al., 2005; Lehtinen et al., 2011). The eCSF of avians (chick) and mammals (rodents and human) displays a dynamic expression pattern of hundred of proteins including essential growth and survival factor for the developing brain (Parada et al., 2005, 2006; Zappaterra and Lehtinen, 2012). Indeed, the main constituents of eCSF are proteins whose enrichment is 30-fold higher at embryonic stages than in the adult cerebrospinal fluid (Birge et al., 1974; Dziegielewska et al., 1980). Proteomic analysis of eCSF revealed the presence of several factors related to cell differentiation or proliferation such as fibroblast growth factors (Martin et al., 2006), insulin-like growth factors (Salehi et al., 2009), sonic hedgehog (Huang et al., 2010), Wnts (Lehtinen et al., 2011), lipoproteins (Parada et al., 2008), and nerve growth factor (Mashayekhi et al., 2009). While most of these proteins have a serous origin (Gato et al., 2004); some of them are directly secreted into the eCSF, such as Shh (Huang et al., 2009) or IGF-1 which are produced by the choroid plexus (Salehi et al., 2009).

Although the analysis of eCSF has gained recent attention as a promising avenue in the success of neuronal stem cell technology (Zappaterra and Lehtinen, 2012), the factors responsible for its effects are only beginning to be uncovered. One possible candidate as an eCSF morphogenetic molecule is SCO-spondin. This high molecular mass glycoprotein is secreted to the eCSF by the subcommissural organ (SCO), a highly conserved brain gland present throughout the vertebrate phylum (Rodriguez et al., 1992, 1998; Meiniel and Meiniel, 2007). The SCO is one of the first structures to differentiate in the chick brain, expressing SCO-spondin as early as the third day of development (Didier et al., 2007; Caprile et al., 2009). This structure is located at the dorsal diencephalic-mesencephalic boundary, which, according to the prosomeric model, corresponds to the roof plate of prosomere 1, underneath the posterior commissure (PC). The SCO is composed of radial glial cells whose apical domains face the third ventricle and, hence, contact the cerebrospinal fluid, whereas their basal domains extend single processes that cross the nerve bundles of the PC and are attached to the pial membrane (Sterba et al., 1982; Rodriguez et al., 1992, 1998).

In spite of the fact that the sequence of SCO-spondin was reported more than 10 years ago (Didier et al., 2000), its precise function still remains to be elucidated. With respect to its biochemical structure, SCO-spondin is a giant glycoprotein of more than 5000 amino acids that display a multidomain organization, including the presence of several thrombospondin repeats (TSR), low-density lipoprotein receptor type A repeats (LDLrA), EGF-like domains, von Willebrand factor domains (vWF), one emilin (EMI) motif, and a C-terminal cystine knot (CTCK) (Didier et al., 2007). The presence of some of these domains has been reported in other proteins related with neurogenesis like thrombospondin 1 or reelin (Adams and Tucker, 2000; Panteri et al., 2006; Lu and Kipnis, 2010).

SCO-spondin is secreted apically, to the cerebrospinal fluid, as well as basally, toward the extracellular matrix contacting the axons of the PC (Caprile et al., 2009). The best characterized route of SCO-spondin secretion is toward the cerebrospinal fluid where it aggregates and forms the Reissner's fiber (RF); a thread-like dynamic structure that grows caudally from the SCO through the fourth ventricle and the central canal of the spinal cord, where it is finally degraded at the level of the *ampulle caudalis* (Molina et al., 2001). The RF has been proposed to regulate CSF production, composition, and circulation (Cifuentes et al., 1994; Rodriguez and Yulis, 2001; Caprile et al., 2003). However, the appearance of RF occurs several days after the onset of SCO-spondin secretion, indicating that, at least during this period, this protein remains soluble in the eCSF. The possible SCO-spondin neurogenic role during early development is suggested by *in vitro* experiments, where solubilized RF or peptides derived from the SCO-spondin sequence promote the survival (Monnerie et al., 1997) and differentiation (El Bitar et al., 2001) of neuronal cells.

Considering the early secretion of SCO-spondin, its biochemical structure, and the neurodifferentiation effect observed *in vitro*, we hypothesized that SCO-spondin affects the behavior of neuroepithelial cells during early brain development. To test this hypothesis, we used a loss of function approach in chick embryos by injecting and electroporating a SCO-spondin-specific shRNA expression vector into the neural tube. Our results show that SCO-spondin is crucial for PC formation and for proper brain development. The absence of this protein generates an increase in neuroepithelial cells division *in vivo*, showing ectopic cellular cluster in the diencephalon and mesencephalon, at the expense of cellular differentiation toward the neuronal lineage. The long-range mode of action of this protein is further supported by *in vitro* experiments, in which mesencephalic explants cultured in SCO-spondin depleted eCSF leads to a dramatical reduction of neurodifferentiation and an increase in mitosis of neuroepithelial cells.

## Materials and methods

### Chick embryos

Fertilized chick eggs were incubated at 38°C in a humidified incubator for specific time intervals. Embryos were staged according to Hamburger and Hamilton (1992). Experiments were conducted following the guidelines outlined in the Biosafety and Bioethics Manual of the National Commission of Scientific and Technological Research (CONICYT, Chilean Government) and the Ethics Committee of the University of Concepción.

### Immunohistochemistry

Embryos were fixed for 24 h in Carnoy, dehydrated in ascending concentrations of alcohols and embedded in paraplast. Brains were oriented to obtain 5–7 μm thick sagittal sections of prosomere 1. Sections were immunostained with mouse monoclonal primary antibodies raised against vimentin and NCAM cytoplasmic domain (H5 and 4D, respectively, from Developmental Studies Hybridoma Bank, University of Iowa, Iowa City, IA) as well as with a rabbit anti Reissner's fiber glycoproteins antibody (AFRU) that recognizes SCO-spondin (Caprile et al., 2009), a mouse anti-βIII tubulin antibody (clone Tuj1, Promega, Madison, WI, USA) and an anti-proliferating cell nuclear antigen (PCNA, PC10 ab29 Abcam). Antibodies were diluted in Tris-HCl buffer containing 1% bovine serum albumin (TRIS-BSA). Goat anti-mouse Alexa-546 and anti-rabbit Alexa-488 antibodies (Invitrogen, Carlsbad, CA) were diluted 1:100 in TRIS-BSA and incubated for 2 h at room temperature. Nuclei were visualized with TOPRO-3 (Invitrogen, Carlsbad, CA). For peroxidase staining, sections were incubated with a secondary goat anti-rabbit IgG coupled to peroxidase (Jackson Immunoresearch, West Grove, PA) diluted 1:100 in the same buffer. Images were acquired with a laser confocal Nikon Eclipse TE2000-U microscope.

The immunohistochemistry of mesencephalic explants was made following the same protocol and using anti-BrdU (G3G4, Developmental Studies Hybridoma Bank, University of Iowa, Iowa City, IA), anti cleaved caspase-3 (ASP175, Cell Signaling Technology), and anti-βIII tubulin (clone Tuj1, Promega, Madison, WI, USA) antibodies.

### Scanning electron microscopy

Stage HH18 Chick embryos were fixed for 2 h by immersion in 2.5% glutaraldehyde buffered to pH 7.4 with 0.1 M phosphate. After manually performing a sagittal cut through the midline of the brain, the tissue was dehydrated in ethanol until critical-point drying, ion covered with gold, and examined with an Etec microscope (Etec Corp., Hayward, CA) (del Brio et al., 2000).

### eCSF extraction

eCSF from stages HH23-HH34 embryos was obtained as previously described (Gato et al., 2004) with small modifications. In order to avoid contamination with neuroepithelial cells, eCSF was gently sucked up under the dissecting microscope with a glass micro-needle, carefully introduced into the middle of the mesencephalic cavity. To minimize protein degradation, eCSF samples were kept at −15°C with a protease inhibitor cocktail (Sigma P2714), aliquoted, and frozen at −80°C until used.

### Western blot

For immunoblot analysis, 25 μg of total proteins were extracted from stage HH23–34 eCSF or from DMEM (Sigma) conditioned with stage HH36 subcommissural organ explants. Samples were fractionated by electrophoresis in 3%–15% linear gradient sodium dodecyl sulfate polyacrylamide gels and subsequently electrotransferred onto nitrocellulose membrane in a buffer containing 25 mM TRIS-HCl, pH 8.3, 192 mM glycine, 0.2% SDS and 20% methanol, at 25 mA, for 14 h (Towbin et al., 1979). Non-specific protein binding sites were blocked by incubating the nitrocellulose membranes with 5% non-fat milk in 0.1 M phosphate buffered saline buffer containing 0.1% Tween-20, for 2 h at room temperature. Membranes were probed with the AFRU antibody (1:15,000) overnight. Anti-IgG rabbit secondary antibodies (1:5000) (Jackson Immunoresearch) were incubated for 2 h at room temperature. Immunoreactive proteins were detected with an enhanced chemiluminescence system (SuperSignal, Pierce, Rockford, IL), as instructed by the manufacturer.

### Plasmid construction

The shRNA-SCO-spondin plasmid was constructed using the kit siSTRIKETM U6 Hairpin Cloning System- hMGFP (Promega, Madison, WI). The shRNA for SCO-spondin was designed using the programs www.promega.com/sirnadesigner and www.rnaiweb.com/RNAi/siRNA_Design. Oligonucleotide sequences were as follows (5′ to 3′): shRNA-SCOspondin-Forward ACC GGA CAG AGC AGG TAA CAG ATT CAA GAG ATC TGT TAC CTG CTC TGT CCT TTT TC; shRNA-SCOspondin-Reverse TGC AGA AAA AGG ACA GAG CAG GTA ACA GAT CTC TTG AAT CTG TTA CCT GCT CTG TC; Scrambled-Forward ACC GGA AGA CCG AAA CGG TAA GTT CAA GAG ACT TAC CGT TTC GGT CTT CCT TTT TC; Scrambled-Reverse TGC AGA AAA AGG AAG ACC GAA ACG GTA AGT CTC TTG AAC TTA CCG TTT CGG TCT TC. These oligonucleotides were annealed and ligated into the siSTRIKE-hMGFP vector and the ligation was confirmed by PstI (NEB Inc.) digestion. Oneshot® Top10 (Invitrogen, Carlsbad, CA) cells were transformed with the resulting plasmids and grown in LB media (MO BIO Laboratories, Inc. Carlsbad, CA, USA) in the presence of ampiciline 1 mg/ml (USBiological, Swampscott, MA). Plasmid purification was made with HiSpeed Plasmid Maxi Kit (Quiagen GmbH, Hilden, Germany).

### Culture and transfection of SCO-cells

Cultured glial SCO cells were obtained from HH34 chick embryos. Briefly, the prosomere 1 roof plate was dissected and digested for 10 min with trypsin 0.12% (wt/vol) in phosphate buffer 0.1 M (pH 7.4, 320 mOsm) and further triturated to homogeneity with a fire-polished Pasteur pipette. 100,000 cells per well were plated onto glass coverslips in a 12-wells plate and incubated for 3 days with Dulbeco's Modified Eagle's Medium (DMEM) supplemented with 10% fetal bovine serum, penicillin and streptomycin. Transfection of shRNA-SCOspondin was carried using Lipofectamine 2000 (Invitrogen) following the manufacturer's instructions. The expression of SCO-spondin was analyzed by immunohistochemistry. The transfection of the plasmid was corroborated by the expression of GFP. Images were acquired with a laser confocal Nikon Eclipse TE2000-U microscope.

### Injection and electroporation of shRNA-SCO-spondin *in ovo*

The injection and electroporation *in ovo* was performed as described in Krull (2004) with some modifications. Briefly, the neural tube of HH 9–11 embryos was injected with 1 mg/ml plasmid DNA containing 0.1% Fast Green (Sigma) for visual monitoring of the injection. Several drops of chick Ringer's solution were dropped onto the embryo after DNA injection. Electrodes were placed above (cathode) and below (anode) the diencephalon. Conditions used for electroporation were five Squarewave electrical pulses of 25 V, 50 ms pulse length, using the Ovodyne electroporator TSS20 (Intracel, Royston Herts, UK) and platinum electrodes. Following manipulation, the eggs were sealed with Parafilm (American National Can™, Greenwich, CT) and returned to the incubator. Twenty-four to thirty-six hours after electroporation, GFP expression was analyzed and embryos displaying expression at the level of the dorsal diencephalon were returned to the incubator until harvesting at HH29.

### Organotypic cultures of mesencephalic neuroectoderm

Organotypic cultures of HH20 optic tecta were performed as described by Gato et al. (2005) and maintained at 37°C with 5% CO2 for 24 h in the presence of 0.01 mM 5-Bromo-2′-deoxyuridine (BrdU, Sigma) and one of the four following media: (1) DMEM (Sigma), (2) SCO-spondin positive conditioned medium obtained from the supernatant of HH36 SCO organ cultures maintained for 4 days in DMEM, (3) 80% DMEM with 20% stage HH25 eCSF, and (4) 80% DMEM with 20% stage HH25 eCSF and supplemented with a 1:300 dilution of the AFRU anti SCO-spondin antibody. After 24 h, the explants were fixed in paraformaldehide 4% and processed for immunohistochemistry to monitor proliferation (anti-BrdU antibody), apoptosis (anti-caspase 3 antibody), and neuronal differentiation (anti-βIII tubulin antibody). The positive areas of explants stained with each antibody as well as the total explant area were analyzed with the Image J program. Error bars represent s.e.m. and statistical analyses were performed using the Student's *t*-test. Differences were considered significant for *p* < 0.05.

## Results

### SCO-spondin is present in the eCSF from early stages of development

**Figure 1:**
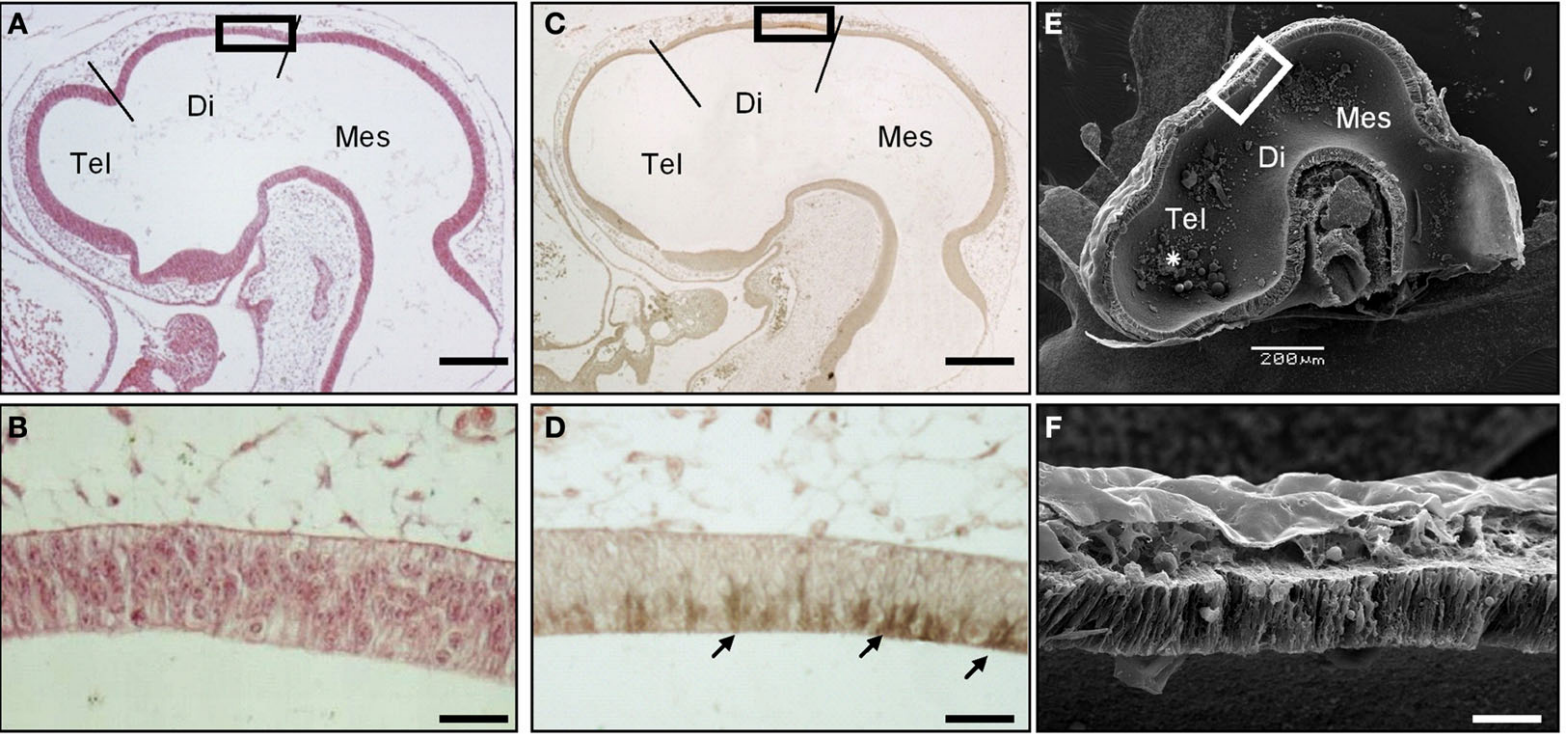
**SCO-spondin is expressed in the SCO at early developmental stages.** Sagittal sections of stage HH17 chicken brain. **(A–B)** Hematoxilin-Eosin staining. **(C–D)** Immunohistochemistry with anti-SCO-spondin showing the presence of this protein in the apical region of caudal diencephalic roof plate (arrows in **D**). **(E–F)** Scanning electron microscopy showing the presence of aggregates within the brain cavities (asterisk in **E**). **(B,D,F)** Higher magnification of the diencephalic roof plate region boxed in **(A,C,E)**, respectively. At this early developmental stage, while the diencephalic roof plate does not present any marked morphological difference from the rest of the neuroepithelium, it strongly expresses SCO-spondin. Tel, Telencephalon; Di, Diencephalon; Mes, Mesencephalon; Scale bars represent 200 μm in **(A,C,E)**; 25 μm in **(B,D,F)**.

To precisely describe the spatiotemporal expression pattern of SCO-spondin, we performed immunohistochemical staining on chick embryo sections from early developmental stages. SCO spondin was first detected at stage HH17 in the diencephalic roof plate (Figures 1A–D) where it was restricted to the apical domain, suggesting its secretion to the eCSF (arrows in Figure 1D). At this stage, the diencephalic roof plate is similar to the rest of the neuroepithelium, consisting of a pseudostratified epithelium, whose basal and apical domains contact the eCSF and the external limiting membrane, respectively (Figures 1E–F).

**Figure 2:**
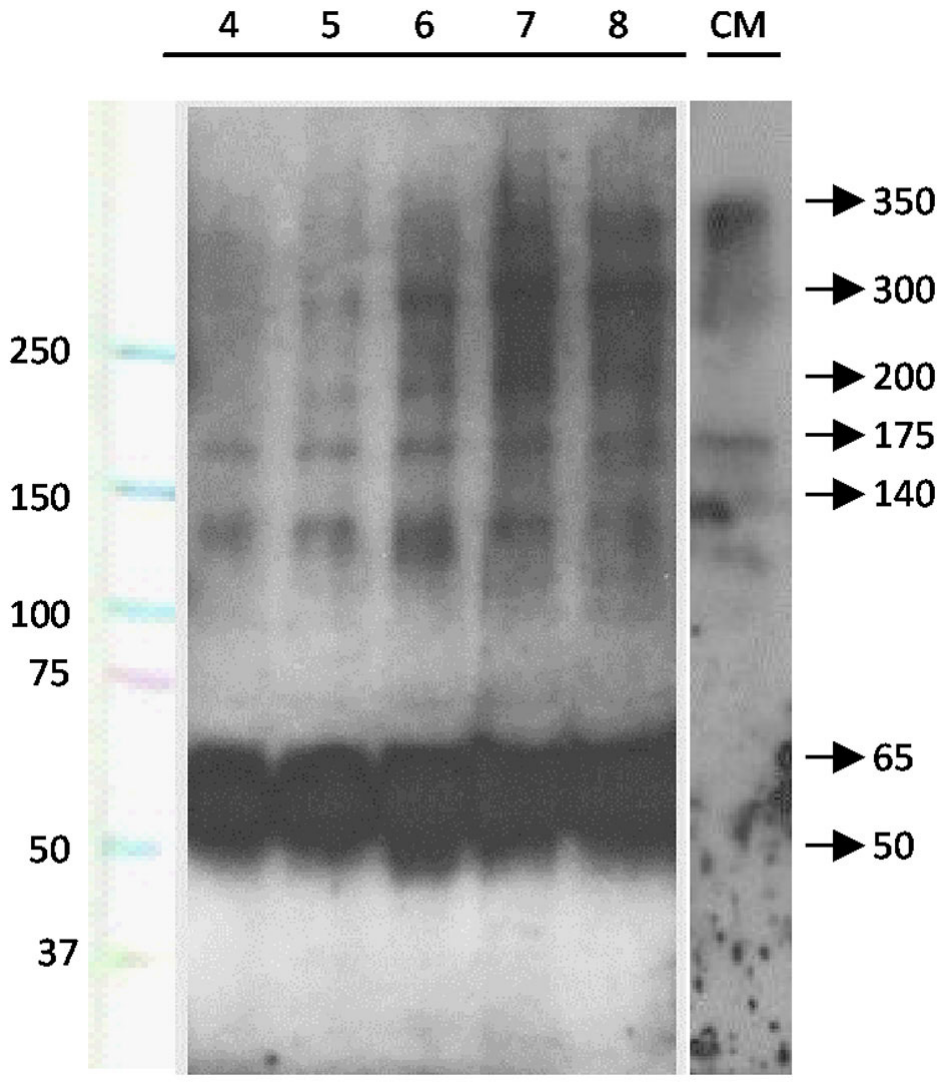
**SCO-spondin is present in the eCSF as early as the fourth day of development.** Lanes 4–8: western blot analyses performed with anti-SCO-spondin antibody on eCFS extracted from chick embryos at stages comprised between the fourth and eighth day of development (HH23 to HH34). CM: western blot analyses performed with anti SCO-spondin antibody on conditioned medium obtained from HH36 SCO explants cultured in DMEM for four days. Lane 1 indicates molecular weight standards. From the fourth day of development onward, the eCSF contains four AFRU-immunoreactive bands of 175, 140, 65, and 50 kDa. Larger bands of 200, 300, and 350 kDa appear progressively during the subsequent days. Note the presence of the 140 and 175, 200, 300, and 350 kDa bands in the conditioned medium.

The possible secretion of SCO-spondin to the eCSF was confirmed by western blot performed on eCSF from chick embryos at different stages of development (Figure 2). The results show that at HH23 (fourth day of development) the anti SCO-spondin recognizes four bands of 175, 140, 65, and 50 kDa; while at later stages additional bands of 350, 300, and 200 kDa appear, which is in agreement with previous reports (Hoyo-Becerra et al., 2006; Vio et al., 2008). Similar bands are found in the conditioned medium from HH36 SCO explants, with the exception of the smaller bands of 65 and 50 KDa (Figure 2, CM lane).

### SCO-spondin binds to the apical domain of neuroepithelial cells *in vivo*

**Figure 3:**
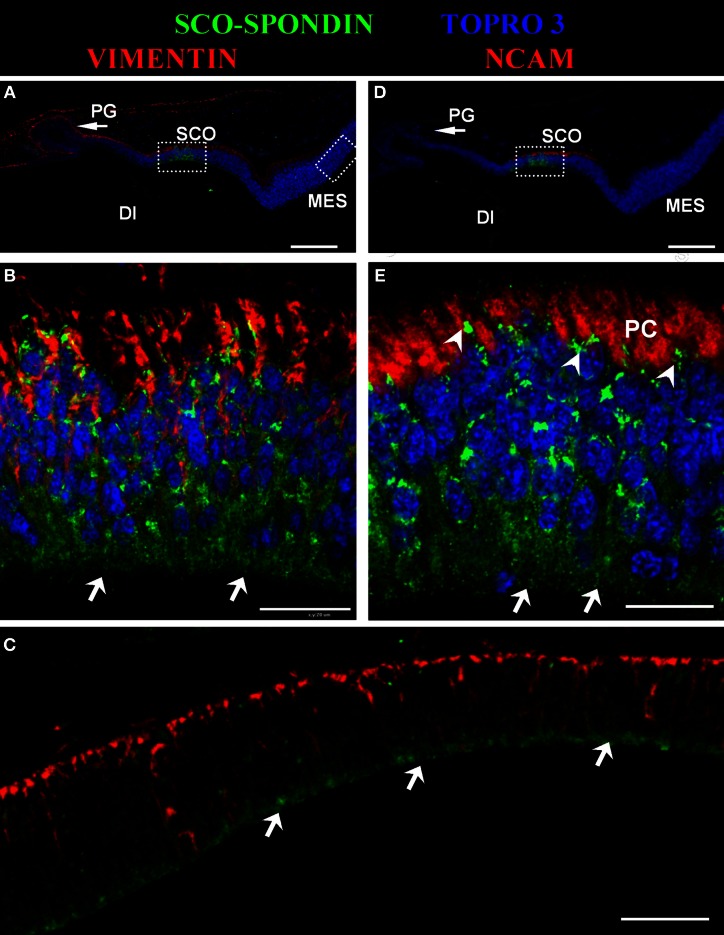
**Secreted SCO-spondin diffuses posteriorly and binds to the apical membrane of the mesencephalon.** Sagittal sections of dorsal diencephalon and mesencephalon of HH23 chicken embryos. **(A–C)** Immunohistochemistry with antibodies against SCO-spondin and vimentin counterstained for nuclei with TOPRO-3. **(B)** Higher magnification of the diencephalic area boxed in **(A)**, showing the localization of SCO-spondin in the cells of diencephalic roof plate (SCO). **(C)** Higher magnification of the mesencephalic area boxed in **(A)**, showing the localization of SCO-spondin at the level of the apical membrane of the mesencephalic neuroepithelium (arrows in **C**). **(D–E)** Immunohistochemistry with antibodies against SCO-spondin and NCAM counterstained for nuclei with TOPRO-3. **(E)** Higher magnification of the area boxed in **(B)** showing the localization of SCO-spondin in contact with the axons of the posterior commissure (arrowheads in **E**) and in the apical region of the SCO cells in contact with the eCSF (arrows in **B** and **E**). Di, Diencephalon; Mes, Mesencephalon; PC, Posterior Commissure; PG, Pineal Gland; SCO, subcommissural organ. Scale bars represent 100 μm in **(A,C,D)**; 200 μm in **(B,E)**.

**Figure 4:**
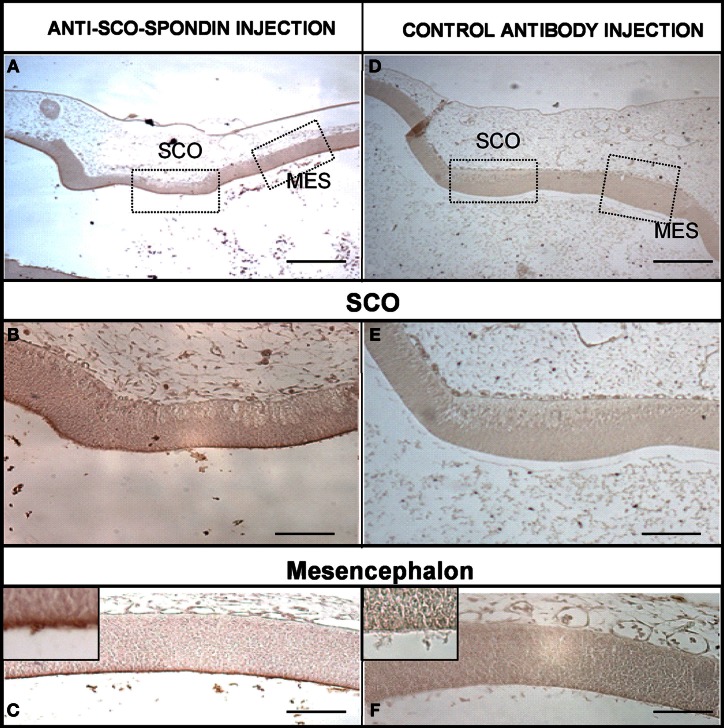
**SCO-spondin is bound to the neuroepithelium apical membranes *in vivo*.** Sagittal sections of dorsal diencephalon and mesencephalon of HH27 chicken embryos. **(A–C)** Embryos were injected with anti–SCO-spondin antibody and left to develop for 24 h before being sacrified and immunostained using anti-rabbit IgG. Area boxed in **(C)** shows the presence of a thin immunoreactive line at the level of the neuroepithelial cells apical membrane. **(D–F)** Control experiment with an unrelated antibody. Scale bars represent 400 μm in **(A,D)**; 150 μm in **(B,E)**; 200 μm in **(C,F)**.

The presence of SCO-spondin in the eCSF of early chick embryos led us to investigate if this glycoprotein interacts with the apical side of neuroepithelial cells. For this purpose, we realized immunohistochemistry with anti SCO-spondin on sectioned HH23 chick brains embryos (Figure 3). The results show that the immunoreaction is exclusively localized to the cells bodies present in the diencephalic roof plate (Figures 3A,B,D,E). At this stage the immunoreaction covers the entire cell, including the apical region in contact with the eCSF (arrows in Figures 3B,E) as well as the basal region in contact with the NCAM-positive axons of the PC (arrowheads in Figure 3E). The rest of the neuroepithelium is immunonegative for SCO-spondin, except for a thin line covering the apical region of mesencephalic apical membrane (arrows in Figure 3C) and a weak signal observed in the medial and basal part of the mesencephalic cells. In order to confirm the SCO-spondin binding to the neuroepithelial apical membrane, the SCO-spondin antibody was injected to the eCSF of live HH24 embryos. After 24 h, animals were sacrificed and the localization of the SCO-spondin antibody was assessed with anti-rabbit IgG. Our results confirm the binding of anti-SCO-spondin to the apical membrane of neuroepithelial cells *in vivo* (Figures 4A–C). The negative control, where unrelated antibodies were injected in the same way showed no immunoreaction (Figures 4D–F).

### *In ovo* inhibition of SCO-spondin

**Figure 5:**
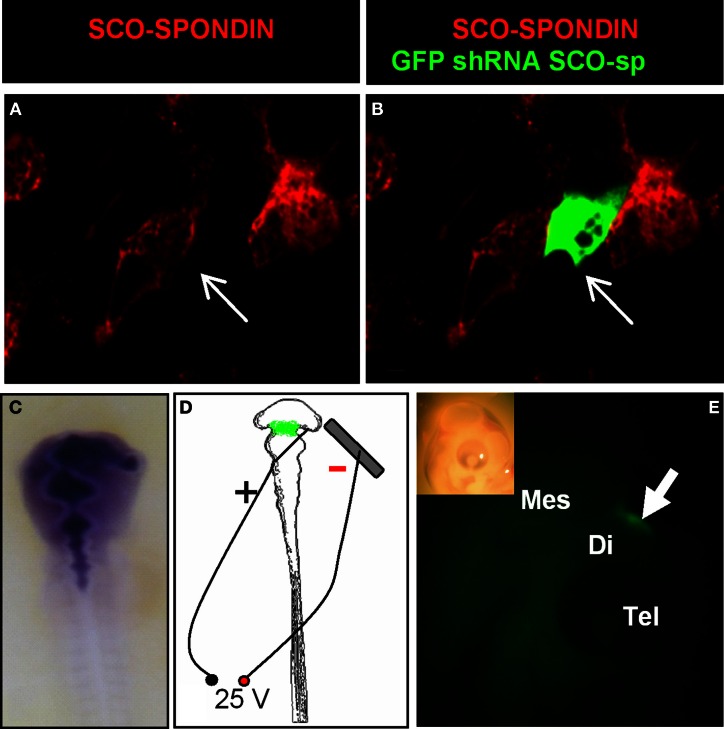
**Efficiency of the SCO-spondin shRNA expression plasmid and standardization of the electroporation procedure. (A–B)** Primary culture of HH34 SCO cells were transfected with SCO-spondin shRNA plasmid and immunostained for SCO-spondin. Note that GFP-positive transfected cells are immunonegative for SCO-spondin (arrows in **A–B**). **(C)** Co-injection of the SCO-spondin shRNA expression plasmid with Fast Green into the neural tube at HH11. **(D)** Schematic drawing showing the respective position of the positive electrode on the dorsal diencephalic region and negative electrode below the embryo. **(E)** Lateral view of a chick embryo head 24 h post-electroporation (inset) showing GFP expression at the level of the dorsal diencephalic- mesencephalic boundary (arrow in **E**).

**Figure 6:**
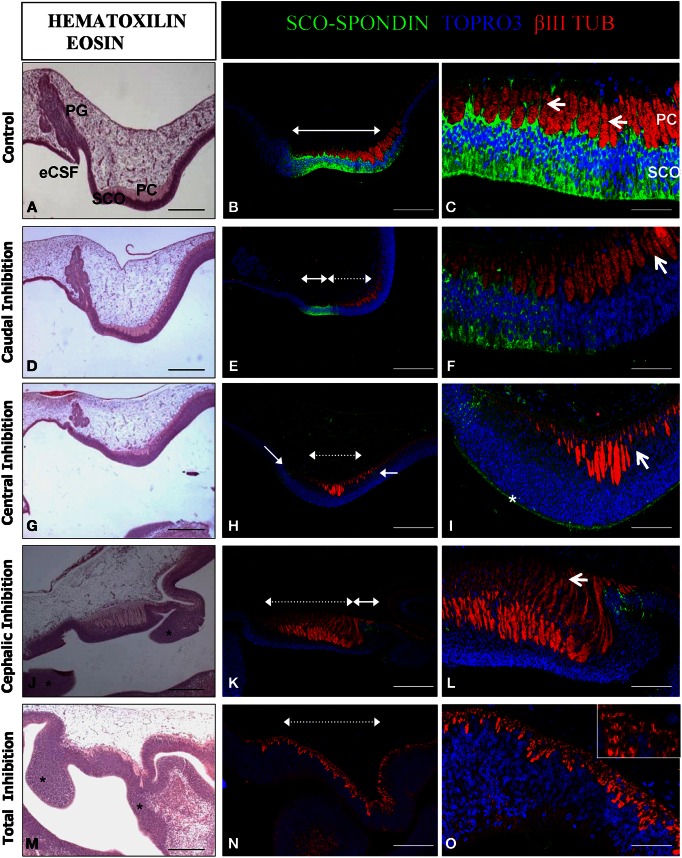
**Effect of the SCO-spondin loss of function on diencephalic development.** Sagittal sections of dorsal diencephalon of HH29 chick embryos with partial or total inhibition of SCO-spondin, and stained with hematoxilin-eosin **(A,D,G,J,M)** or with antibodies against SCO-spondin and βIII tubulin and counterstained with TOPRO3 **(B,C,E,F,H,I,K,L,N,O). (A–C)** Control embryos. **(D–O)** Embryos with partial or total inhibition of SCO-spondin expression. **(D–F)**, Caudal inhibition; **(G–I)**, central inhibition; **(J–L)**, cephalic inhibition; and **(M–O)**, complete inhibition of SCO-spondin. In **(B,E,H,K,N)** dotted double arrowheads show the inhibited region, while plain double arrowheads show remnants of SCO-spondin expression. Arrows in **(C)** show the basal prolongations of the SCO cells. Arrows in **(F,I)** point at the presence of nuclei between the axonal fascicles, showing that the basal prolongations have been substituted by cell bodies. Asterisk in **(I)** shows the presence of SCO-spondin in contact with the apical membrane of the diencephalon neuroepithelial cells. Asterisks in **(J,M)** show ectopic cellular clusters located at the dorsal diencephalon. Note the absence of a recognizable pineal gland in **(J,M)**. Arrow in **(L)** shows dramatic axonal defasciculation. The inset in **(O)** shows that a normal axonal tract is absent and has been replaced by βIII tubulin positive neurons. PG, Pineal gland; SCO, Subcommissural organ; PC, Posterior commissure; eCSF, Embryonic cerebrospinal fluid. Scale bars represent 250 μm in **(A,B,D,E,G,J,K,M,N**); 100 μm in **(C,F,I,L,O)**.

**Figure 7:**
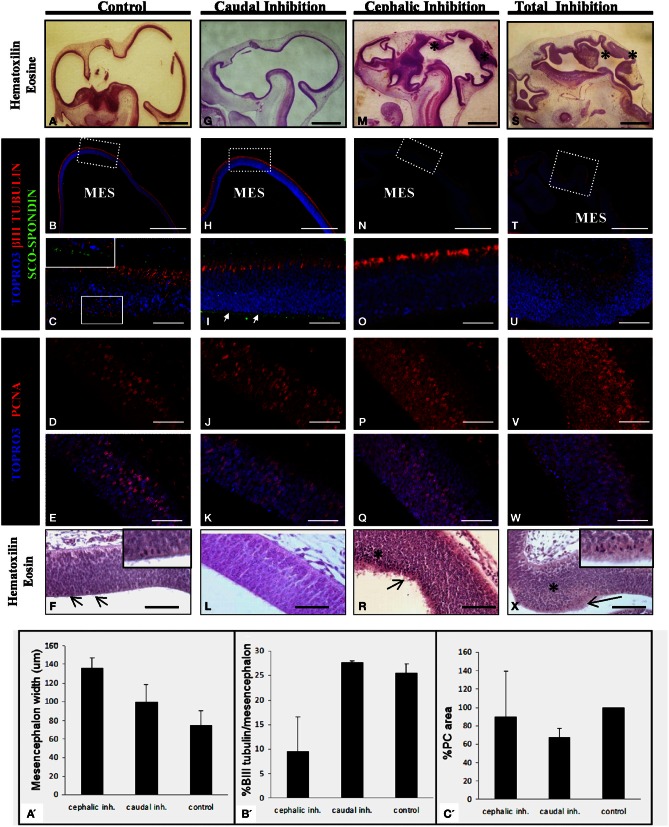
**Effect of the SCO-spondin loss of function on mesencephalic development.** The panels show sagittal sections of mesencephalon of HH29 chick embryos with partial or total inhibition of SCO-spondin. **(A–F)**, Control embryos; **(G–L)**, caudal inhibition; **(M–R)**, cephalic inhibition; **(S–X)**, complete inhibition of SCO-spondin; **(A,F,G,L,M,R,S,X)**, Hematoxilin-Eosin staining; **(B,C,H,I,N,O,T,U)**, Immunohistochemistry for β III tubulin and SCO-spondin counterstained with TOPRO3. Inset in **(C)** and arrows in **(I)** show the apical localization of SCO-spondin. **(D,E,J,K,P,Q,V,W)** Immunohistochemistry for PCNA counterstained with TOPRO3. Asterisks show ectopic cellular bodies in **(M,S,R,X)**. **(A′–C′)** Quantification of the phenotypes observed in the SCO-spondin inhibited animals revealing differences in mesencephalic width **(A′)**, percentage of βIII-tubulin positive area with respect to total mesencephalic area **(B′)**, and PC area **(C′)**. Scale bars = 2 mm in **(A,B,G,H,M,N,S,T)**; 50 μm in **(C–F,I–L,O–R,U–X)**.

In order to analyze the function of SCO-spondin *in ovo* during early CNS development, we designed a plasmid allowing the co-expression of GFP with a SCO-spondin specific shRNA or with a control scrambled shRNA. The high efficiency of the shRNA was first confirmed on primary culture of chick SCO-cells expressing SCO-spondin, showing that even though the number of transfected cells was low, all of them were immunonegative for SCO-spondin (Figures 5A,B). After the injection of the vector into the neural tube of HH11 embryos (Figure 5C), electroporation of the diencephalic roof plate was performed by placing the positive electrode at the dorsal diencephalic region and the negative electrode beneath the embryo (Figure 5D). One day after electroporation, the GFP expression was monitored in order to ensure that the expression of the plasmid occurred in the accurate region (Figure 5E), and the selected embryos were left to develop until stage HH29 before being examined. From the total of the electroporated embryos, nine of them displayed GFP expression at the level of the diencephalic roof plate and survived until stage HH29. Immunohistochemical analysis with the anti-SCO-spondin antibody revealed that these animals can be separated into two different groups according to their phenotypes: (1) animals inhibited at the level of the cephalic region of the SCO. This group includes animals with complete inhibition and others expressing SCO-spondin exclusively at the level of the caudal region, and (2), animals in which SCO-spondin is specifically and exclusively inhibited at the level of the caudal or the central region of the SCO. Below, we will describe the consequences of these different types of inhibition on the development of the diencephalon (Figure 6) and the mesencephalon (Figure 7).

The diencephalon developed normally in animals that received the scrambled shRNA. Such control animals present a SCO-spondin positive region of 700 μm in length occupying an area of approximately 9800 μm^2^, located precisely below the PC (Figures 6A–C). Under normal conditions, cells of the SCO extend basal prolongations that emerge from the cell body, traverse the PC, dividing this axonal tract in fascicles, and attach to the external limitant membrane (arrows in Figure 6C). The size of the PC fascicles grows progressively in rostro-caudal direction, being smaller at the rostral region. Hence, the length of the basal prolongations of the SCO cells is variable and closely correlates with the thickness of the PC fascicles. At this stage, the primordium of the pineal gland is present at the level of the dorsal diencephalon, rostraly to the SCO (Figure 6A). The diencephalon of animals in which SCO-spondin is inhibited caudally (*n* = 3) presents a diminution of 32 ± 9% in the PC area, which nevertheless displays a normal degree of axonal fasciculation, and a pineal gland primordium similar to the control (Figures 6D–F). We obtained one animal showing inhibition at the level of the central region, whose phenotype was similar to animals inhibited caudally with a small fasciculated PC, and presence of a normal pineal gland primordium (Figures 6G–I). In such animal, SCO cells have lost their radial morphology and present nuclei in the entire thickness of the SCO. Remarkably, while central inhibition abrogates SCO-spondin expression in the cell bodies, this protein is still strongly detected on the apical side of the SCO cells (asterisk in Figure 6I). Cephalic inhibition of SCO-spondin (Figures 6J–L) does not affect the PC area but causes a higher grade of axonal defasciculation (arrow in Figure 6L). Such animals (*n* = 3) lack the pineal gland primordium and show ectopic cellular clusters located in the dorsal diencephalon (asterisks in Figure 6J). Likewise, animals with complete inhibition (*n* = 2, see Figures 6M–O) lack a pineal gland and exhibit ectopic cellular cluster in the dorsal diencephalon (asterisks in Figure 6M). Additionally, complete inhibition causes a drastic diminution in the number of PC axons which are replaced by βIII tubulin positive cell bodies (inset in Figure 6O).

With respect to the mesencephalic region, the optic tectum of WT and control animals that received the scrambled shRNA display a normal and homogeneous thickness of 75±15μm which positively stains for βIII tubulin in its dorsal-most border (Figures 7A–C). The level of cell proliferation was revealed by the presence of a nuclear immunostaining of PCNA in 31 ± 4.3% of the cells (Figures 7D–E). Histological analysis reveals a discrete presence of mitotic spindles on the cells contacting the cerebrospinal fluid (arrows and inset in Figure 7F). The apical border of these cells consists of an homogeneous, uninterrupted, epithelium, and presents immunoreaction for anti SCO-spondin (inset in Figure 7C). Animals in which SCO-spondin was inhibited in the caudal region of the SCO (Figures 7G–L) present a normal optic tectum with a SCO-spondin immunoreactivity at the level of the apical surface (arrows in Figure 7I) and a level of βIII tubulin (Figure 7H–I), and PCNA (Figures 7J–K) immunoreaction similar to the control embryos. By contrast, the general mesencephalic morphology was severely affected in embryos with total inhibition, or in embryos displaying an inhibition localized to the cephalic region of the SCO (Figures 7M–X). Such embryos present a thicker neuroepithelial wall, including the presence of numerous undifferentiated cells (asterisk in Figures 7M,S). βIII tubulin immunoreactivity is highly reduced, particularly in animals with total inhibition (Figure 7U) and the PCNA immunoreactivity is dramatically increased and is present in both the cell nucleus and the cytoplasm. This localization of PCNA has been described in other proliferative cell types, during the M phase of the cell cycle (Iwao et al., 2005). In agreement with these results, hematoxilin-eosin staining shows a dramatic increase in the number of mitotic cells of mesencephalic neuroepithelium (see arrows in Figures 7R,X, and inset in **7X**). Furthermore, the apical border of mesencephalic cells is irregular, with the presence of detached cells (Figures 7R,X), and has lost its immunoreactivity for SCO-spondin (Figures 7O,U).

In summary, the animals with a SCO-spondin cephalic inhibition have a wider mesencephalon than controls (136 ± 11 μm vs. 75 ± 15μm, Figure 7A′), and also display a smaller area staining positively for βIII tubulin (9.5 ± 7% vs. 25.6 ± 1.7%, Figure 7B′). By contrast, animals with inhibition of SCO-spondin at the level of the caudal region show a mesencephalon similar to control animals concomitantly with a reduced PC area (67.7 ± 9% compared to control animals, Figure 7C′).

### Effect of SCO-spondin on mesencephalic explants

**Figure 8:**
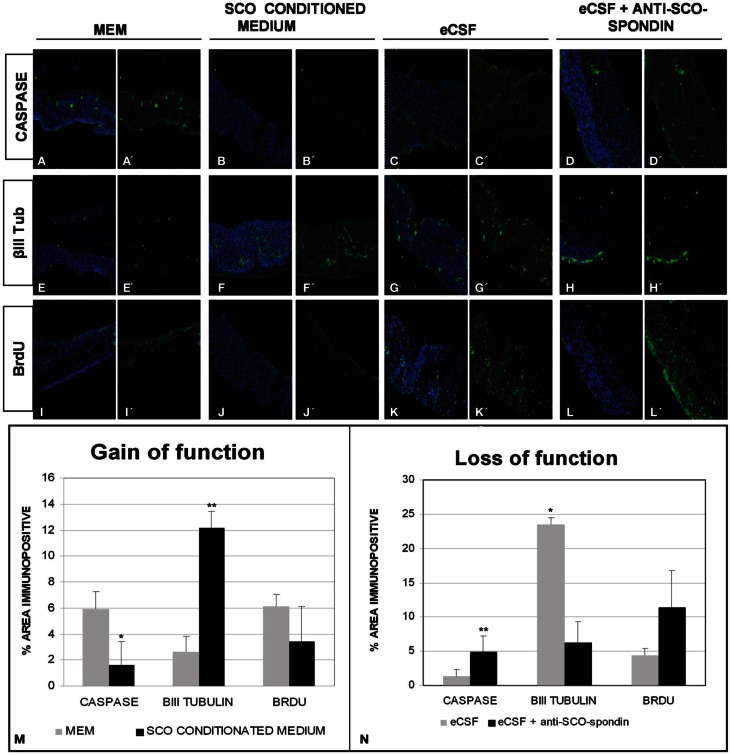
**SCO-spondin regulates the behavior of mesencephalic cells *in vitro*.** Optic tectum explants from HH20 embryos cultured in presence of DMEM **(A,E,I)**; SCO conditioned DMEM **(B,F,J)**; DMEM supplemented with 20% eCSF **(C,G,K)**; DMEM supplemented with 20% eCSF and incubated with anti SCO-spondin antibody **(D,H,L)**. The explants were analyzed for the presence of activated caspase 3 **(A′–D′)**, βIII tubulin **(E′–H′)** and BrdU incorporation **(I′–L′)**. Panels **(A–L)** show the merge with the TOPRO3 nuclear signal used to counterstain the tissue. **(M–N)** Quantification of the area immunopositive for the different antibodies in each experiment. ^*^p < 0.05; ^**^p < 0.01. Bars mean ± SEM.

The mesencephalic malformations found in animals in which SCO-spondin was inhibited led us to investigate the long-range function of this protein *in vitro*, using optic tecta explanted from HH20 chick embryos (Figure 8). On the one hand, we analyzed the effect of SCO-spondin gain of function by comparing DMEM with conditioned DMEM medium that has been in contact with SCO explants that secrete SCO-spondin (Figure 2 CM). On the other hand, we performed loss of function experiment by comparing normal eCSF with SCO-spondin-depleted eCSF. The gain of function experiment revealed that conditioned medium from SCO-explants produces a fivefold increase in neurodifferentiation (2.6 ± 1.2 vs. 12.15 ± 1.2; see Figures 8E–F) a threefold decrease in apoptosis (5.92 ± 1.3 vs. 1.6 ± 1.7; see Figures 8A–B) and a diminution in proliferation (6.04 ± 0.9 vs. 3.4 ± 2.6; see Figures 8I–J). Additionally, SCO-spondin inhibition generates a threefold increase in apoptosis (1.2 ± 0.6 vs. 4.8 ± 2.3; see Figures 8C–D) and proliferation (4.4 ± 1.8 vs. 11.4 ± 5.3; see Figures 8K–L), as well as a fourfold decrease in neurodifferentiation (23.4 ± 3.8 vs. 6.2 ± 3.09; see Figures 8G–H). Taken together, these *in vitro* results are similar to the *in vivo* situation, where the inhibition of SCO-spondin generates an increment in the mesencephalic proliferation at the expense of neurodifferentiation.

## Discussion

**Figure 9:**
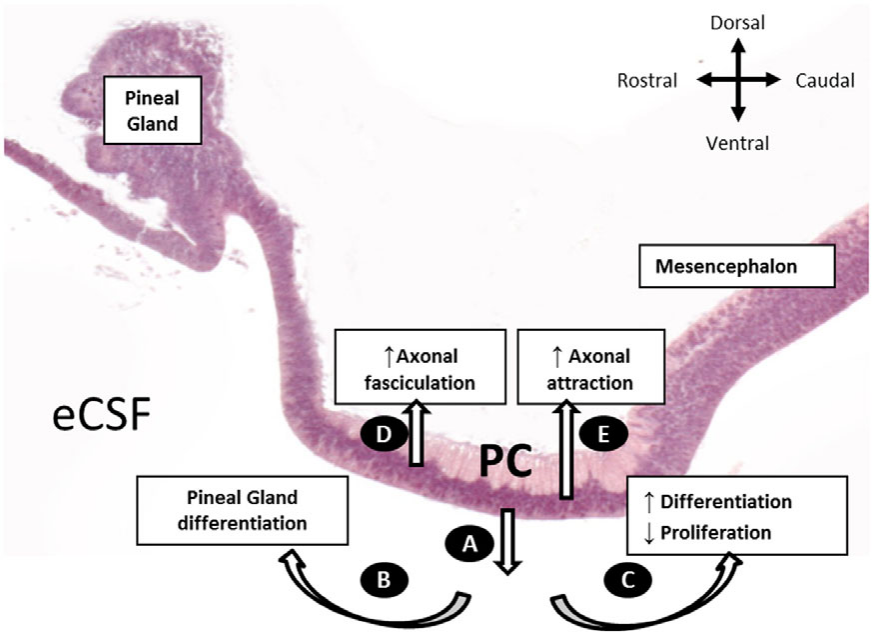
**Schematic representation of the SCO-spondin secretory routes and their respective roles, as proposed in the present work. (A)** Apical secretion of SCO-spondin to the eCSF. **(B)** Rostral spreading of SCO-spondin promoting pineal gland differentiation. **(C)** Caudal spreading of SCO-spondin towards the mesencephalic cavity, inducing neurodifferentiation at the expense of proliferation. **(D)** Basal secretion of SCO-spondin by the rostral SCO cells, promoting axonal fasciculation of the PC. **(E)** Basal secretion of SCO by the caudal SCO cells promoting incorporation of new axons to the PC.

In this study, we performed, for the first time, an *in vivo* inhibition of SCO-spondin expression. Furthermore, by targeting the inhibition to different regions of the diencephalic roof plate, we showed that SCO-spondin is a pleiotropic protein, fulfilling different functions according to its secretion mode (Figure 9). When apically secreted, SCO-spondin remains soluble in the eCSF (Figure 9A) and binds to the apical membrane of neuroepithelial cells, thereby affecting their differentiation and proliferation, while its basal secretion at the level of the PC seems to contribute to the fasciculation and attraction of the PC axons (Figures 9D,E). These results are in agreement with previously reported *in vitro* experiments in which either SCO-spondin or peptides derived from its sequence promote fasciculation (Stanic et al., 2010), neurite outgrowth (Meiniel et al., 2003; Stanic et al., 2010), and differentiation (Monnerie et al., 1997; El Bitar et al., 2001).

### Basally secreted SCO-spondin regulates PC formation

The following previous lines of evidence have led some authors to propose that SCO-spondin contributes to the PC development (Meiniel et al., 2008; Caprile et al., 2009; Hoyo-Becerra et al., 2010; Stanic et al., 2010; Grondona et al., 2012): (1) the concomitant formation of SCO and PC, (2) the similarity of SCO-spondin with other molecules involved in axonal guidance, (3) the early secretion of this protein toward the extracellular matrix surrounding the PC axons, and (4) *in vitro* experiments where the addition of SCO-spondin or peptides derived from its sequence increase neurite length and fasciculation. Here, we provide direct *in vivo* evidence that SCO-spondin is crucial for PC formation, as its loss of function either causes a marked decrease in the number of axons (animals with total inhibition), a moderate diminution in the number of axons (inhibition at the caudal region), or axonal defasciculation (animals with cephalic inhibition). The different roles observed for SCO-spondin when it is expressed in the cephalic region (fasciculation) and caudal region (incorporation of new axons) could be due to the steep SCO-spondin rostro-caudal expression gradient (Stanic et al., 2010). In this respect it is interesting to note that the presence of integrin β1 (the hypothetical SCO-spondin receptor) in the axonal membrane is negatively correlated to the concentration of its ligand (Condic and Letourneau, 1997). Hence, it is tempting to propose that, in the caudal region, the lower local concentration of SCO-spondin will promote the formation of integrin/SCO-spondin complexes, leading to axonal outgrowth and incorporation of new axons to the PC. According to this model, a higher availability of SCO-spondin in the cephalic region will induce the internalization of surface integrins, diminishing the interaction between the axons and their surrounding extracellular matrix, and, in turn, favoring the interaction between neighboring axons (i.e., fasciculation), a process mediated by axonal adhesion molecules, such as NCAM (Van Vactor, 1998).

### Apically secreted SCO-spondin remains soluble in the eCSF and binds neuroepithelial cells

The apical secretion of SCO-spondin to the CSF and its polymerization to form the RF during late development and adulthood, are widely accepted. However, the presence of a functional and soluble form of SCO-spondin in the eCSF is a matter of recent studies (Hoyo-Becerra et al., 2006; Vio et al., 2008). Our work reveals that from the third day of chick development onward, SCO-spondin is secreted to the eCSF and that it remains soluble at least until day 8. We also provide evidence showing that this protein is firmly bound to the apical membrane of neuroepithelial cells at HH24 (fourth day of development), since it is recognized by SCO-spondin antibodies injected to the eCSF *in vivo*. Our results open new questions regarding the biochemical structure of the soluble form of SCO-spondin detected in the eCSF and to the directionality of its diffusion at this stage. By performing western blots on HH23 eCSF, we have detected the presence of four bands of 175, 140, 65, and 50 kDa; while at later stages additional bands of 350, 300, and 200 kDa appear. The presence of similar molecular weight bands was found in the CSF of 7 days postnatal rats (Vio et al., 2008) using the same antibody (AFRU) as well as the anti-P15 antibody raised against a peptide derived from the bovine SCO-spondin. These observations suggest the existence of several SCO-spondin isoforms generated by alternative splicing and/or by cleavage. This possibility is in agreement with the presence of several transcripts detected by northern-blot using an SCO-spondin-specific probe (Meiniel et al., 2003).

A smaller 138 kDa human SCO-spondin isoform has been reported (A2VEC9-2, Uniprot), containing eight LDLR-A, two EGF-like and three TSP domains, but lacking the CTCK domain, responsible for oligomerization. Therefore, it remains possible that the 140 kDa SCO-spondin isoform detected in the eCSF at early developmental stages (Figure 2) correspond to this small isoform, and that the onset of expression of larger isoforms containing the CTCK triggers polymerization and RF formation after the seventh day of development (Schoebitz et al., 1986; Caprile et al., 2009).

### Region specific morphogenetic role of SCO-spondin

The present work reveals a strong region-specific effect for SCO-spondin, as complete or cephalic inhibition severely affects mesencephalic development, while animals with caudal inhibition display an almost normal morphology.

These results suggest that the region of SCO-spondin inhibition is more important than the total area of inhibition, since the presence of few SCO-spondin immunopositive cells in the cephalic region is sufficient to sustain a normal mesencephalic development. While it is possible that the SCO-spondin secreted at the caudal and cephalic region may correspond to distinct isoforms with different roles on PC and mesencephalic development, we favor a second hypothesis according to which the SCO-spondin secretion pathway differs between the caudal and cephalic region. Indeed, we found that animals whose SCO-spondin expression is restricted to the cephalic region display an SCO-spondin immunoreactivity in the apical region of mesencephalic cells. In contrast the mesencephalic cells of animals that express SCO-spondin only at the caudal region are devoid of this immunoreactivity.

The secretion of SCO-spondin to the eCSF opens the question about how this protein will spread into the brain cavities The circulation of eCSF at early stages of development is not yet fully understood, since the absence of choroid plexus does not provide the cephalo-caudal directionality of liquid flows observed in the adult. In this respect, recent studies performed in living *Xenopus leavis* embryos report the existence of a semicircular fluid flow in the telencephalic and mesencephalic cavities, acting the cerebral aqueduct (the region that contacts the SCO and where SCO-spondin is secreted) as a bridge between the eCSF of both cavities (Mogi et al., 2012). The diencephalic roof plate is therefore a favored region whose secretions can efficiently spread into the brain cavities, since they will be carried away both anteriorly (e.g., toward the pineal gland) and posteriorly (e.g., toward the mesencephalic cavity).

### SCO-spondin as a morphogen carrier?

One fundamental issue that still remains to be tackled is the molecular mode of action of SCO-spondin. Our *in vitro* experiments show that the addition of a SCO-spondin inhibitory antibody diminishes drastically the ability of native eCSF to promote neurodifferentiation (Figure 8). It is known, however, that the eCSF contains a variety of factors involved in brain development such as dystroglycan, retinoic acid, FGF2, or LDL (Gato and Desmond, 2009; Zappaterra and Lehtinen, 2012) suggesting that these factors might influence each other or act redundantly. For instance, more than 60% of the neural differentiation activity exerted by native eCSF requires the presence of LDL (Parada et al., 2008). Interestingly, to fulfill this role, LDL requires the presence of others eCSF components that still remain to be identified (Parada et al., 2008). Considering the presence of several LDLR-A domains in SCO-spondin, it is possible that SCO-spondin is involved in the delivery of lipoproteins to neuroepithelial cells. In addition to their function as lipid carriers, LDLR-A domains can also act as carriers for morphogens of the hedgehog (Hh) and Wnt families (Panakova et al., 2005; Willnow et al., 2007). The association between SCO-spondin and lipoproteins-morphogens could offer an efficient mean to transport them around the whole brain cavities, and to increase the local concentration of such morphogens. Indeed, if this turned out to be the case, each morphogens will be presented as multiple copies on the same lipoprotein particle, generating a multivalent ligand complex able to promote homomeric clustering of their cognate receptors, as well as heterodimeric interaction between different morphogens. Furthermore, the presence of multiple domains in SCO-spondin like TSP, and EFG-like would increase the range of combinatorial interactions between extracellular ligands.

In summary, our work strengthens the idea that SCO-spondin is a multifunctional protein, involved locally in PC development, and also able to exert a long-range function on remotely located regions of the brain. The secretion and diffusion of a soluble form of SCO-spondin into the eCSF allows its binding to the apical surface of the neuroepithelial cells of the diencephalon and mesencephalon, where it triggers signaling events promoting the neuronal differentiation and exit of mitosis. Future challenges will involve deciphering the molecular actors collaborating with SCO-spondin, such as morphogens, receptors, and signaling pathways.

## Grant sponsor

FONDECYT; Grant number: 1110723 (T. Caprile).

### Conflict of interest statement

The authors declare that the research was conducted in the absence of any commercial or financial relationships that could be construed as a potential conflict of interest.
